# Dynamic, multiphase magnetic resonance imaging of in vivo physiological processes with long-lived hyperpolarized ^15^N,d_9_-betaine

**DOI:** 10.1126/sciadv.adx8417

**Published:** 2025-07-02

**Authors:** Ingeborg S. Skre, Magnus Karlsson, Juan Diego Sánchez-Heredia, Rie B. Olin, Mathilde H. Lerche

**Affiliations:** ^1^ Center for Hyperpolarization in Magnetic Resonance, Department of Health Technology, Technical University of Denmark, Kgs. Lyngby, Denmark.; ^2^ Technical University of Cartagena (UPCT), Cartagena, Spain.

## Abstract

Hyperpolarized magnetic resonance imaging (HypMRI) offers valuable insights into dynamic physiological processes in vivo. However, the short signal lifetime of hyperpolarized ^13^C-labeled compounds commonly used in HypMRI studies restricts investigations to fast molecular reactions and rapid distributions. Here, we introduce hyperpolarized ^15^N,d_9_-betaine (trimethyl-^2^H_9_-^15^N-glycine) as an endogenous MRI contrast agent with a long-lived signal suited for comprehensive molecular tracking. With in vivo detectability exceeding 14 minutes and high polarization efficiency, ^15^N,d_9_-betaine supports both real-time and delayed-phase MRI from a single dose, enabling flexible, multistage imaging. In preclinical models, renal ^15^N,d_9_-betaine images were acquired with strong signal-to-noise ratios across resolutions. This extended imaging window facilitates tracking molecular distribution, assessing tissue perfusion, and monitoring cellular uptake relevant to betaine’s roles in cellular protection. By extending MRI capabilities across timescales, hyperpolarized ^15^N,d_9_-betaine holds promise for applications like organ function assessment, disease monitoring, and real-time evaluation of therapeutic interventions, advancing noninvasive molecular imaging.

## Teaser

Long-lived hyperpolarized ^15^N,d_9_-betaine enables flexible MRI across time to track molecular distribution and organ function.

## INTRODUCTION

Accurate, noninvasive imaging of kidney function remains a major clinical and research challenge. In nephrology, assessments rely heavily on global markers such as glomerular filtration rate, urinary sediment, and proteinuria or on invasive kidney biopsies that provide only localized, single time point information. These limitations hinder efforts to understand solute handling and tissue-level physiology in real time and complicate the development and monitoring of targeted interventions (*1*, *2*). While molecular imaging holds promise for noninvasive visualization of renal processes, there is a specific need for physiologically relevant imaging agents that can report on solute distribution, osmolyte accumulation, and renal perfusion dynamics across multiple timescales.

Betaine (trimethylglycine) is an endogenous, highly soluble, zwitterionic quaternary ammonium compound that serves critical biochemical functions in humans (*3*). It is obtained through dietary intake or synthesized endogenously from choline via irreversible oxidation (*4*, *5*). Betaine has two primary roles: First, it acts as an osmoprotectant, protecting cells from osmotic stress, particularly in the kidneys, where it may accumulate at extraordinary concentrations (>100 mM) to counteract rapidly fluctuating electrolyte and urea levels (*3*, *6*, *7*). Second, betaine functions as a key methyl donor in one-carbon metabolism, supporting the methionine cycle by donating a methyl group to homocysteine—a toxic metabolite (in elevated concentrations) and a cardiovascular risk factor—which is then further converted to methionine (*8*, *9*).

The biological and clinical significance of betaine extends beyond these roles. Elevated urinary excretion of betaine and increased plasma homocysteine levels have been linked to diseases such as cardiovascular disorders, liver dysfunction, and neurological conditions (*10*–*16*). Conversely, dietary supplementation with betaine has been shown to improve kidney, liver, cardiovascular, and neurological health (*5*, *9*, *10*, *17*). Moreover, betaine is US Food and Drug Administration approved for the treatment of homocystinuria, a rare metabolic disorder characterized by excessive homocysteine accumulation (*18*, *19*). Given its multifaceted roles, noninvasive imaging of betaine’s distribution and dynamics in vivo holds substantial potential for providing insight into both physiological processes and disease pathophysiology.

To date, no in vivo imaging studies of betaine have been reported using noninvasive molecular imaging techniques. Choline, the endogenous precursor of betaine, has been extensively studied with [^11^C] and [^18^F] positron emission tomography (PET) imaging, although these studies primarily focus on choline’s role in cancer metabolism through its involvement in phospholipid synthesis rather than betaine production (*20*–*23*). Fluorinated choline analogs are substrates for choline kinase but are not recognized by enzymes responsible for choline oxidation, and thus no fluorinated derivatives of betaine have been observed in [^18^F] PET studies (*23*). Conversely, [^11^C]betaine has been detected as a metabolite of [^11^C]choline in red blood cells in humans and rats (*24*).

^13^C hyperpolarized magnetic resonance imaging (HypMRI) has gained ground in clinical research as an alternative, noninvasive approach to PET. It allows real-time tracking of metabolic and physiological processes, with a higher temporal resolution (1 to 5 s) (*25*) compared to PET studies, which predominantly rely on static imaging, acquired several minutes posttracer administration (*26*–*30*). In particular, hyperpolarized ^13^C-pyruvate has demonstrated clinical utility in oncology for monitoring metabolic conversion to ^13^C-lactate (*31*–*33*), and ^13^C-urea has been used for renal function and perfusion imaging (*34*–*40*). However, ^13^C HypMRI is fundamentally limited by the short spin-lattice relaxation times (*T*_1_) of ^13^C nuclei, restricting the imaging window to 1 to 2 min postinjection. 1-^13^C, d_2_ betaine (^13^C-betaine) has also been investigated as an enzymatic product in a hyperpolarized ^13^C-choline in vitro study (*41*). However, like other ^13^C-labeled compounds, hyperpolarized ^13^C-betaine is constrained by a short signal lifetime, with a *T*_1_ estimated to be 30 s at 3 T in vitro (*41*), thereby confining its use to early uptake phases. As a result, its potential for studying delayed dynamics and biodistribution is limited. These temporal constraints highlight the need for innovative approaches to extend imaging windows and capture both early and late physiological processes.

Hyperpolarized ^15^N-labeled compounds offer a promising solution to these challenges. Quaternary ^15^N nuclei exhibit substantially longer *T*_1_ relaxation times compared to ^13^C, enabling prolonged signal lifetimes suitable for multiphase imaging (*42*). Several hyperpolarized ^15^N compounds have been investigated for molecular imaging (*43*). The *T*_1_ of ^15^N-choline in buffer has been reported to range from 4 to 9 min, depending on field strength and degree of deuteration (*44*, *45*). In blood and in vivo, the *T*_1_ of ^15^N-choline has been reported to decrease to ~2 to 3 min at high field (*46*, *47*). Similarly, hyperpolarized l-^15^N,d_9_-carnitine has shown a long *T*_1_ of almost 3 min in vivo (*48*). While these endogenous compounds have demonstrated potential for spectroscopic imaging, their limitations in achieving physiological relevance and resolving metabolic pathways restrict their broader utility in in vivo studies. The utility of ^15^N-choline is constrained by its toxicity profile, which prevents the administration of the high millimolar concentrations needed for achieving physiological distribution. On the other hand, ^15^N-carnitine, as an endogenous energy storage molecule, is expected to be well-tolerated at high concentrations. However, its in vivo signal lifetime is insufficient for late time point biodistribution studies. Furthermore, neither ^15^N-choline nor ^15^N-carnitine provides adequate chemical shift separation to resolve distinct metabolic pathways relevant for their biochemical roles. Notably, Shchepin *et al.* (*49*) demonstrated that multi-^15^N-labeled metronidazole can be hyperpolarized, achieving ~16% ^15^N polarization and an exceptionally long in vitro ^15^N *T*_1_ of nearly 10 min. Building on this, Guarin *et al.* (*50*) translated hyperpolarized [^15^N_3_]metronidazole to in vivo MRI, observing ^15^N signal persistence in rat brain for more than 1 min. These advances underscore the promise of long-lived ^15^N molecules as contrast agents, enabling metabolic and physiological imaging on timescales beyond the reach of conventional ^13^C molecular contrast agents.

By leveraging the extended *T*_1_ of hyperpolarized quaternary ^15^N nuclei and combining it with the biological relevance of betaine, this study seeks to overcome current temporal constraints in molecular imaging and advance its capabilities.

Here, we investigate hyperpolarized ^15^N,d_9_-betaine (trimethyl-^2^H_9_-^15^N-glycine), a novel molecular imaging agent that combines the biological importance of betaine with a prolonged signal lifetime. By substituting the methyl protons of betaine with deuterium, we achieve a long *T*_1_, enabling signal detection for more than 14 min in vivo. This innovation overcomes the temporal constraints of traditional hyperpolarized ^13^C imaging, facilitating both rapid dynamic imaging of early uptake and delayed-phase imaging of late biodistribution. Moreover, the long signal lifetime and high polarization achieved for ^15^N,d_9_-betaine allow for multiple imaging experiments from a single hyperpolarized dose. This unique capability enables comprehensive assessments of physiological processes, including molecular distribution and tissue perfusion in a single imaging session. Combined with betaine’s expected good safety profile as a widespread endogenous compound, these features suggest that ^15^N,d_9_-betaine may become a versatile tool for metabolic and physiological investigations.

## RESULTS

### Long *T*_1_ under various conditions and high polarization of ^15^N,d_9_-betaine

To evaluate the hyperpolarization performance of ^15^N,d_9_-betaine, its *T*_1_ under different chemical and magnetic conditions and its polarization build-up using high-field dynamic nuclear polarization (DNP) were assessed. Repeated low flip angle magnetic resonance spectroscopy (MRS) experiments were performed to measure *T*_1_ in buffer and blood at 3 and 9.4 T, at ~37°C, Table 1.

**Table 1.** *T*_1_ of ^15^N,d_9_-betaine under different in vitro conditions. The *T*_1_ was measured in buffer at both 9.4 T (*n* = 3) and 3.0 T (*n* = 3), as well as in blood at 3.0 T (*n* = 4).

Medium	*T*_1_ (min)	*B*_0_ (T)	*T* (°C)
Buffer (*n* = 3)	6.9 ± 0.2	9.4	37
Buffer (*n* = 3)	7.0 ± 0.2	3.0	~35
Blood (*n* = 4)	7.1 ± 0.3	3.0	~35

The *T*_1_ of ^15^N,d_9_-betaine measured 7 min in vitro and remained remarkably stable across varying magnetic fields and chemical environments. The apparent magnetic field independence of the *T*_1_ relaxation can be attributed to the highly symmetrical arrangement around the ^15^N position. The symmetry of quaternary ammonium present in structures like betaine, choline, and carnitine makes the chemical shift anisotropy (*51*), which strongly scales with the field, a less important contributor to the *T*_1_ relaxation. This extended relaxation time permits flexible imaging windows to capture both early dynamic uptake and delayed-phase biodistribution.

In vivo, ^15^N,d_9_-betaine signals were observable at all investigated time points up to 14 min, and the apparent *T*_1_ measured in the abdominal region of rodents at 3 T was 5.6 ± 0.6 min (*n* = 3), likely influenced by physiological factors such as tissue clearance, distribution, and metabolism (in vivo spectra and fits are shown in fig. S1).

**Fig. 1.**
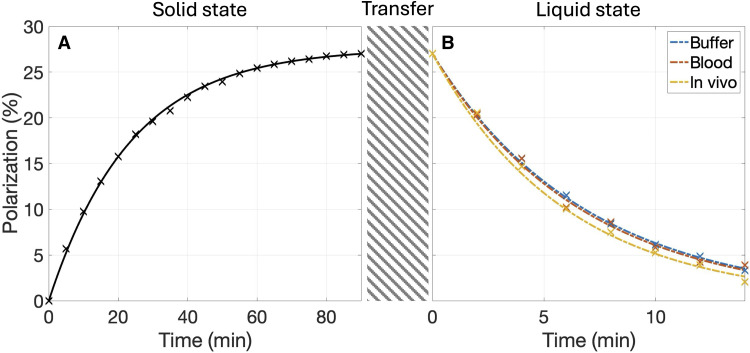
Representative examples of hyperpolarization of ^15^N,d_9_-betaine. (**A**) Solid-state polarization build-up at 6.7 T and 1.25 K. Fit is shown as a solid line and peak integrals as crosses. Maximum solid-state polarization is based on liquid-state polarization measurements. (**B**) Signal decay measurements at 3 T in buffer, blood, and in vivo from the rodent abdominal region. Fits are shown as stippled lines and peak integrals as crosses.

With an optimized DNP protocol at 6.7 T and 1.3 K, a high polarization level for ^15^N,d_9_-betaine was achieved, using a trityl radical as the electron paramagnetic agent. Notably, 27 ± 2.5% polarization (*n* = 5) was reached within ~1.5 hours. Representative solid-state polarization build-ups next to liquid state *T*_1_ measurements in different conditions at 3 T and ~37°C are displayed in Fig. 1.

The combination of a prolonged *T*_1_ and high polarization provided a robust hyperpolarized signal suitable for multiple acquisitions within a single imaging session. Having established both *T*_1_ stability and efficient polarization build-up, we next examined the feasibility of rapid dynamic imaging and delayed-phase imaging using ^15^N,d_9_-betaine in vivo.

### Tissue perfusion and molecular distribution from a single hyperpolarized dose

**Fig. 2.**
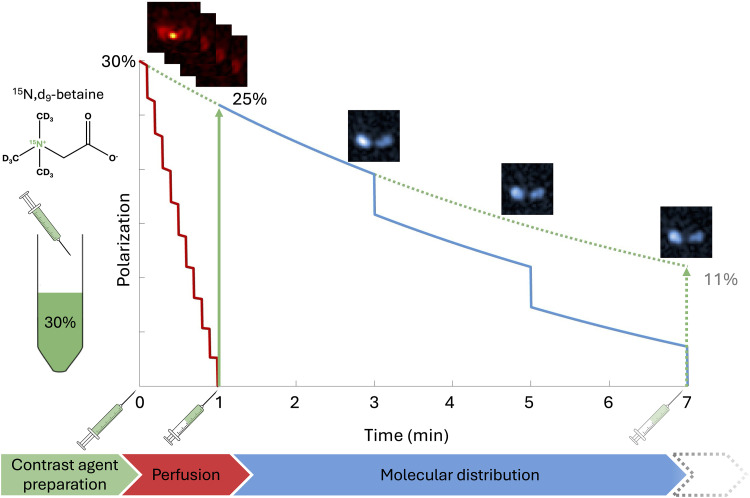
Schematic illustrating the division of a hyperpolarized ^15^N,d_9_-betaine dose for sequential injections. This dual-injection strategy enables both early-phase (perfusion) and late-phase (molecular distribution) imaging with minimal impact on initial polarization. After 7 min, there is still ~11% polarization available, making it possible to plan further imaging experiments as indicated by the stippled arrow in the bottom flow chart. The chemical structure of ^15^N,d_9_-betaine (C_5_H_2_D_9_^15^NO_2_, molecular weight: 127.2 g/mol) is also shown.

To demonstrate the advantages conferred by the robust, long in vitro *T*_1_ of ^15^N,d_9_-betaine, we designed a set of sequential imaging experiments that could be conducted from a single hyperpolarized dose (Fig. 2). Specifically, we divided the hyperpolarized solution into two syringes, enabling two separate injections that each made optimal use of the hyperpolarized signal.

The first injection enables rapid dynamic imaging to capture early uptake kinetics and tissue perfusion patterns with high temporal resolution. Crucially, because the hyperpolarized solution is split into two separate syringes, the signal from the first injection can be fully used through an aggressive pulsing scheme, without affecting the second experiment. Following a short interval, a second injection enables delayed-phase imaging, assessing the late molecular distribution of ^15^N,d_9_-betaine.

This sequential approach leverages the long *T*_1_ and stable polarization of ^15^N,d_9_-betaine, ensuring minimal polarization loss over the delay period and allowing each injection’s pulsing scheme to be tailored to its specific imaging goal. By maintaining a substantial fraction of polarization between injections, signal decay is minimized over the course of both imaging sessions. This enables the capture of maximal information from a single hyperpolarized dose, underscoring the feasibility and efficiency of multiphase imaging with ^15^N,d_9_-betaine. Such multiphase imaging capabilities have implications for studying organ function (e.g., kidney perfusion) within a single experimental or clinical session, thereby reducing total dose requirements and scan times.

### Perfusion and vascular dynamics of ^15^N,d_9_-betaine in rodent kidneys

**Fig. 3.**
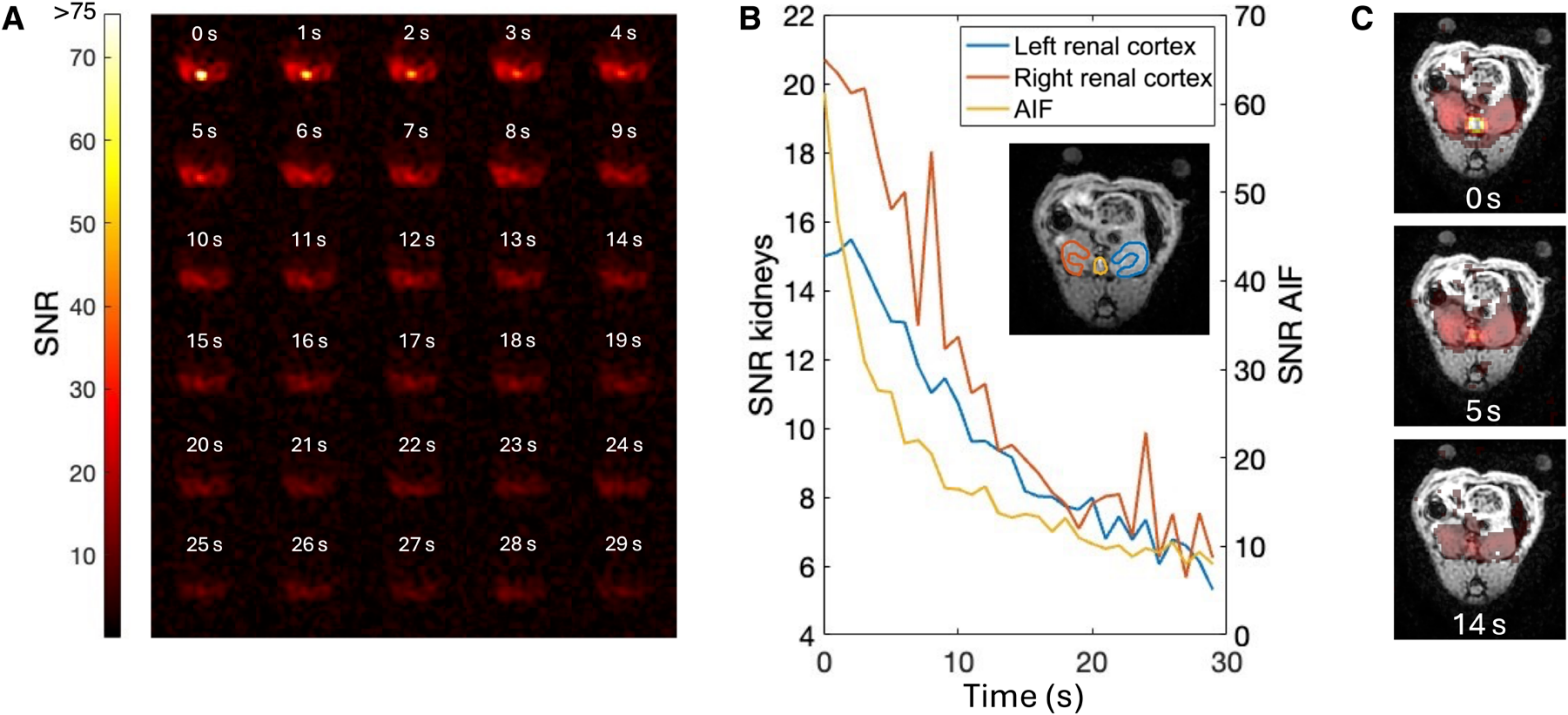
Early dynamic imaging of hyperpolarized ^15^N,d_9_-betaine in rodent kidneys in axial view. (**A**) ^15^N,d_9_-betaine images, windowed at 80% of maximum SNR. (**B**) Time curves of SNR from the vasculature [arterial input function (AIF)] and the left and right renal cortices, as manually delineated from proton scans. The delineated regions of interest are shown on the anatomical reference with colors corresponding to the time curve. (**C**) Selected time points overlayed the anatomical reference. A mask with a threshold of SNR ≥ 5 was applied to the ^15^N,d_9_-betaine images, which share color bar with Fig. 3A.

To capture the uptake and initial distribution of ^15^N,d_9_-betaine in the rodent kidneys, high temporal resolution imaging was initiated immediately after injection and continued at 1-s intervals for 30 s (Fig. 3A). The highest signal initially appeared in the vasculature [signal-to-noise ratio (SNR) ≈ 60], reflecting the direct delivery of ^15^N,d_9_-betaine via the bloodstream. This venous signal rapidly declined, due to radiofrequency (RF) signal depletion, tissue uptake, and bloodstream dilution, while strong localization of the molecular contrast agent in both kidneys became apparent within the first few frames.

Time-intensity curves for the vasculature and the left and right renal cortices are presented in Fig. 3B. Although the renal cortex signals were broadly similar, the right cortex exhibited a slightly higher initial signal and a faster decay, suggesting minor anatomical or physiological differences in local perfusion or uptake. After 30 frames, sufficient signal remained to visualize ^15^N,d_9_-betaine in the kidneys, with an SNR ≈ 6 in the last frame. Collectively, these findings underscore the value of high temporal resolution for elucidating early-phase perfusion dynamics as well as the suitability of ^15^N,d_9_-betaine for rapid in vivo imaging studies.

### Molecular distribution of ^15^N,d_9_-betaine in rodent kidneys

**Fig. 4.**
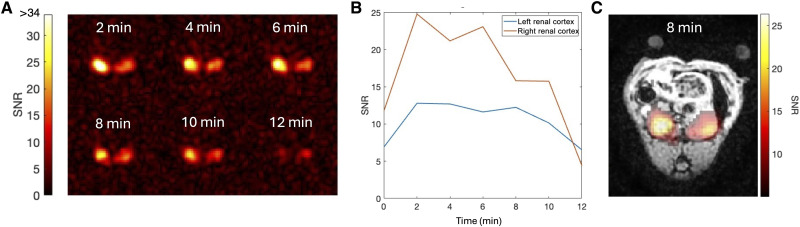
Late dynamic imaging of ^15^N,d_9_-betaine uptake in the rodent kidneys in axial view. (**A**) ^15^N,d_9_-betaine images, windowed at 80% of maximum SNR. (**B**) Time evolution of the SNR in the left and right renal cortices. (**C**) ^15^N,d_9_-betaine image 8 min after injection overlayed the anatomical reference. A mask with a threshold of SNR ≥ 5 was applied to the ^15^N,d_9_-betaine image. The two circles above the rat body are water heating tubes.

To exploit the long, hyperpolarized lifetime of ^15^N,d_9_-betaine for flexible delivery, a second injection was administered immediately after the early dynamic imaging experiment. This experiment, targeted later dynamics by performing imaging every 2 min for 12 min with increasing flip angels, designed to maintain a near-constant SNR throughout the acquisition (Fig. 4, A and B). This approach revealed how ^15^N,d_9_-betaine disseminates and persists in renal tissue, illuminating molecular or clearance processes beyond the initial uptake.

A slight asymmetry was observed between the kidneys, with the right kidney exhibiting ~60% higher SNR than the left and with stable SNR in the interval 2 to 10 min postinjection of 10 to 13 and 16 to 25 for the left and right kidneys, respectively. Nevertheless, the hyperpolarized signal remained sufficiently strong to visualize the distribution of ^15^N,d_9_-betaine 12 min postinjection, corresponding to the seventh frame of the experiment with an SNR of ≈ 6 in a region of interest (ROI) taken over the renal cortex (Fig. 4B). These findings demonstrate the viability of multiphase imaging allowing both rapid uptake and later-phase molecular tissue retention to be studied.

### Higher-resolution imaging at selected time points

**Fig. 5.**
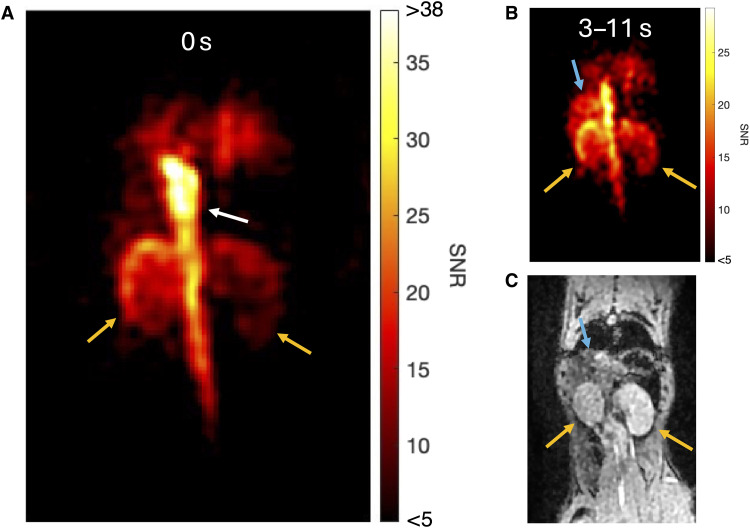
Higher-resolution imaging of ^15^N,d_9_-betaine in the rodent kidneys in coronal view, with an anatomical reference. All five frames are shown in fig. S2. The orange, white, and blue arrows indicate the kidneys, vasculature, and liver, respectively, and are aligned by eye. (**A**) First frame, with color map SNR lower and higher limits of 5 and 38 (80% of max), respectively. (**B**) Sum of frames 2 to 5 (acquired 3 to 11 s postinjection), with a lower color map SNR limit of 5. (**C**) Anatomical reference.

To further demonstrate the versatility of ^15^N,d_9_-betaine for detailed insights into molecular distribution, we performed high-resolution imaging (1.25 mm by 1.25 mm in-plane) at five time points postinjection, Fig. 5. At time point 1, a high-resolution image acquired within 738 ms (Fig. 5A) visualizes the vascular compartments, revealing subtle differences in uptake and perfusion. At later time points (Fig. 5B), the molecular distribution extended into the kidney cortex and liver, highlighting the ability to map betaine across multiple organs.

These findings underscore the potential of combining early-phase dynamic and subsequent high-resolution imaging via separate injections for optimal signal usage, with dynamic data pinpointing the most informative time points for high-resolution acquisitions. This flexibility, allowing for selection of both early- and late-phase high-resolution imaging, further establishes ^15^N,d_9_-betaine as a highly versatile hyperpolarized agent.

## DISCUSSION

We here investigated ^15^N,d_9_-betaine as a hyperpolarized MRI (HypMRI) molecular agent and demonstrated its long *T*_1_ relaxation time and robust hyperpolarized signal, surpassing previously reported ^15^N-labeled compounds investigated in vivo (*43*). With this molecular imaging compound, we showed how both early-phase kidney uptake and late-phase renal tissue distribution could be imaged from a single hyperpolarized dose. We also showed how the molecular signature alternatively could allow for high-resolution imaging in under 1 s, the combination of which could provide application tailored imaging.

Most HypMRI studies use ^13^C-labeled compounds, which benefit from higher MR sensitivity compared to ^15^N, due to a 2.5 times greater gyromagnetic ratio. Imaging of hyperpolarized ^13^C-urea has demonstrated promise in perfusion imaging and is now being evaluated for clinical translation (*35*, *52*, *53*). In rodent studies, renal dynamic imaging of ^13^C-urea has been used to assess perfusion in ischemic reperfusion injury (*54*) and diabetes (*55*) and urea transport in diuretic and antidiuretic states (*56*) and to assess glomerular filtration rates (*57*), using voxel sizes of 1.9 mm by 1.9 mm by 10 mm with a temporal resolution of ~2 s. In comparison, our perfusion imaging experiments with ^15^N,d_9_-betaine used a larger voxel volume of 2.5 mm by 2.5 mm by 15 mm, with a faster 1-s temporal resolution. While ^13^C-labeled agents offer superior early sensitivity, ^15^N,d_9_-betaine enables extended imaging from the same dose, providing complementary molecular information and highlighting the distinct strengths of ^13^C and ^15^N HypMRI.

We acquired ^15^N,d_9_-betaine images in rodent kidneys with a high in-plane resolution of 1.25 mm by 1.25 mm. However, achieving sufficient sensitivity required substantial RF depletion, indicating that higher resolution ^15^N imaging is better suited for selected time points rather than rapid dynamic imaging. By using single time point acquisitions with optimized flip angles amounting to 90° that fully use and deplete the available polarization, a theoretical >200% increase in signal is possible. The *T*_2_ decay constant of ^15^N,d9-betaine is expected to be long, based on reports for structurally related ^15^N-labeled compounds, such as ^15^N-choline, displaying a *T*_2_ of 42 s in aqueous solution (11.7 T) (*44*) and ^15^N,d_9_-carnitine, with a global in vivo *T*_2_ of 4 s (4.7 T) (*48*). A long *T*_2_ decay constant suggests that sensitivity could be further improved with the use of refocused MR sequences, such as steady-state free precession (*58*), which has been successfully applied to high-resolution ^13^C HypMRI of ^15^N,^13^C-urea (*59*). In ^13^C studies using such approaches, an isotropic resolution of 1.2 mm has been achieved for single time point rodent imaging experiments (*39*, *54*).

In addition, the ^15^N transmit-receive RF coil used in this study could be further optimized for rodent imaging. Substantial SNR improvement should be obtained by using an RF coil setup with separate transmit and receive coils. From the transmit side, that would improve B1+ homogeneity, leading to better control of the obtained flip angles. From the receive side, that would allow to use a much smaller coil as a receiver, therefore providing higher sensitivity on the regions closest to the coil. We estimate that for kidney experiments in rodents, an optimized ^15^N coil setup would provide at least a 2.5-fold SNR enhancement.

In clinical practice, PET remains the gold standard for molecular imaging, where ^11^C labeling is particularly useful for imaging of endogenous molecules. PET provides high sensitivity and spatial resolution and usually uses delayed acquisitions to allow for cellular uptake and sufficient tissue contrast. Typically, radiolabel accumulation maps are acquired over several minutes (*28*–*30*), resulting in static images. In contrast, ^15^N,d_9_-betaine enables high-resolution, dynamic molecular mapping on a shorter timescale (<1 s per image), allowing perfusion imaging and motion-artifact reduction through single breath hold or cardiac-gated acquisitions.

Betaine’s organ-specific physiological roles make it a promising candidate for molecular diagnostics, particularly in renal function. Our results highlight betaine’s role as an osmoprotectant in the renal medulla, where it is actively transported by the betaine–γ-aminobutyric acid transporter (BGT1) and dynamically regulated by osmotic conditions (*60*). Altered betaine transport and accumulation may thus serve as an indicator of renal dysfunction, nephron efficiency, or hydration status. Also, betaine’s efficient glomerular filtration and near-complete tubular reabsorption (*61*) support its potential as a biomarker for evaluating renal reabsorption capacity. Increased urinary betaine levels have been associated with chronic kidney disease (*15*) and diabetes (*62*), in humans, supporting its potential as a renal biomarker. Because betaine accumulation is crucial for kidney osmoprotection, hyperpolarized ^15^N,d_9_-betaine could help detect renal pathologies (e.g., diabetic kidney disease) that affect osmolyte handling, by revealing changes in betaine distribution or excretion. In addition, intravenous (IV) betaine has been shown to induce a diuretic effect, with a more pronounced response in hypertensive conditions, suggesting a role in blood pressure regulation (*12*).

Beyond the kidney, betaine is essential in hepatic metabolism, where it functions as a methyl donor in one-carbon metabolism, playing a critical role in lipid regulation (*4*). The liver’s high concentration of betaine transporters suggests that disruptions in hepatic betaine metabolism could be a diagnostic marker for metabolic conditions like non-alcoholic fatty liver disease. Hyperpolarized ^15^N,d_9_-betaine might enable real-time imaging of hepatic one-carbon metabolism, offering a noninvasive window into disorders of methylation chemistry and liver metabolic function. The frequency shift from ^15^N,d_9_-betaine to its primary metabolite dimethylglycine is ~106 Hz at 3 T, which would enable metabolite-specific imaging given sufficient metabolite signal. It should be noted that betaine metabolism is likely to occur on a slower timescale and with smaller metabolite pool sizes than substrates involved in probing energy metabolism, such as hyperpolarized [1-^13^C]pyruvate. Consequently, different acquisition parameters (flip angles and timing) must be specifically optimized for ^15^N,d_9_-betaine when, e.g., investigating one-carbon metabolism in the liver. In the brain and cardiovascular system, betaine stabilizes proteins and modulates homocysteine levels, with potential applications in neurodegenerative diseases and vascular health (*60*).

To enhance ^15^N *T*_1_, we used deuterium-labeled (d_9_) betaine, although deuteration may slightly affect compound behavior. The substitution of deuterium, a stable isotope of hydrogen, changes molecular mass and bond strength, which can influence enzymatic rates (*63*). However, these effects are minor concerning transporter recognition, and BGT1 is thus expected to transport d_9_-betaine similarly to nonlabeled betaine, making it a reliable marker for renal uptake and distribution. Future studies should validate whether d_9_-betaine mimics endogenous betaine distribution, particularly in applications where hepatic metabolism is assessed.

Multi-injection strategies enabled early- and late-phase imaging within a single session; however, high compound loads necessitate toxicity considerations. IV and oral betaine safety can be compared to choline and pyruvate, which have been suggested (^15^N,d_9_-choline) and is commonly used (^13^C-pyruvate) as hyperpolarized molecular agents. Orally administrated betaine demonstrates a high safety margin with an LD₅₀ (lethal dose, 50%) exceeding 11 g/kg comparable to that of pyruvate. However, acute toxicity data for betaine remain limited. The only available reference for IV administration reports moderate toxicity in mice, with an LD₅₀ of ~830 mg/kg (*64*), which falls within the same range as pyruvate (older data suggests >1.25 g/kg in mice) (*65*). In contrast, choline exhibits much higher toxicity, with an LD₅₀ of around 50 mg/kg in mice (*66*). Despite its relatively favorable profile, the potential toxicity of IV-administered betaine should be carefully considered in future studies aiming to optimize dose-response relationships and multi-injection protocols to preserve physiological relevance.

Betaine’s high aqueous solubility (>6.5 M) allows for preparation in vitrifying solutions, a key requirement for dissolution DNP hyperpolarization. This concentration is within the range needed for large-volume dissolution, as recently demonstrated for ^13^C-keto-isocaproic acid (*67*), which shares similar physical properties. These characteristics indicate that scaling up ^15^N,d_9_-betaine sample preparation to clinically relevant doses should be straightforward.

Our study has demonstrated that ^15^N,d_9_-betaine is a versatile, long-lived HypMRI agent that offers important advantages over existing ^13^C-based HypMRI approaches and complements PET imaging. By enabling both early-phase uptake and late-phase tissue distribution from a single dose, ^15^N,d_9_-betaine expands the capabilities of noninvasive molecular imaging. Its capability for multiphase imaging and extended temporal windows enables the acquisition of diverse molecular information across different timescales. Future studies should explore acquisition strategies that leverage the polarization properties of ^15^N,d_9_-betaine, such as serial injections and infusion protocols. Hyperpolarized ^15^N,d_9_-betaine may be used to interrogate other organ systems and disease processes where one-bond carbon metabolism or osmolyte function is relevant. Applications in kidney function, liver metabolism, homocysteine-related disorders, and neurological conditions are promising areas for further investigation, and a deeper understanding of betaine’s organ-specific uptake and clearance in both physiological and pathological states will clarify its utility as a diagnostic and therapeutic tool.

## MATERIALS AND METHODS

### Chemicals

^15^N-glycine, iodomethane-d_3_, and silver(I)oxide were purchased from Merck (Sigma-Aldrich) (Darmstadt, Germany). Trityl radical Ox063 was obtained from GE Healthcare (Chicago, USA). Gadoteridol (active pharmaceutical ingredient of clinical contrast agent ProHance) was obtained from Bracco Imaging (Milan, Italy).

### Synthesis of ^15^N,d_9_-betaine

^15^N-glycine (820 mg, 10.78 mmol) was dissolved in sodium hydroxide (aqueous solution, 12 M, 3.1 ml). Methanol (40 ml) was added to the solution. Iodomethane-d_3_ (6.5 g, 43 mmol) was mixed with methanol (8 ml) and added dropwise to the glycine solution. After addition and stirring for 3 hours, the reaction was checked by ^15^N nuclear magnetic resonance (NMR) showing full conversion to betaine. The solvent was removed using a rotary evaporator, and the remaining solids were dissolved in water (45 ml). Silver(I) oxide (4.4 g, 19 mmol) was added, and the slurry was stirred for 5 min and then centrifuged. The pH of the supernatant, containing the product, was adjusted to approximately 8 with diluted sulfuric acid. This solution was freeze-dried yielding a white solid containing the product and sodium sulfate. The solid was stirred in ethanol (99%, 100 ml). After filtering off the remaining solid (sodium sulfate), the ethanol was evaporated yielding the product as a white powder (1100 mg, 8.6 mmol).

^15^N NMR showed a betaine resonance at 48.9 parts per million (ppm), referenced to ^15^N-glycine at 33.5 ppm (fig. S3). ^13^C{^2^H} NMR displayed resonances at 54.9 ppm (methyl, 3n), 68.4 ppm (methylene, 1n), and 171.8 ppm (carboxyl, 1n) (fig. S4).

### Hyperpolarization and NMR

A polarization medium was prepared by dissolving trityl radical Ox063 (19.3 mg, 13.5 μmol) (GE Healthcare, Chicago, USA) in a 1:1 (v/v) mixture of glycerol and water (155 mg). Gadoteridol was added to this solution [6.8 mg of a solution (100 μmol/g) in water].

Samples for DNP were prepared by dissolving ^15^N,d_9_-betaine (25 mg, 196 μmol or 50 mg, 392 μmol) in polarization medium (25 or 50 mg), yielding approximate concentrations of 50 mM trityl radical and 2.5 mM gadoteridol concentrations, respectively. A lower radical concentration prolonged the polarization build-up time but did not substantially enhance the polarization. The resulting sample amounts, 50 or 100 mg (~45 or 90 μl), were hyperpolarized in a SpinAligner polarizer (Polarize, Kgs Lyngby, Denmark) working at 6.7 T, 1.25 K. The hyperpolarization build-up time constant was ~30 min. After completed build-up, the sample was dissolved in 5 ml in either Dulbecco’s phosphate-buffered saline or in 20 mM phosphate buffer with addition of NaCl for physiologically matched ion strength yielding a 40 mM or 80 mM hyperpolarized ^15^N,d_9_-betaine solution that was used for the MR experiments or for polarization and *T*_1_ measurements on an NMR system.

The polarization level and *T*_1_ of the dissolved betaine were measured at 9.4 T, 310 K on a Bruker Avance Neo spectrometer fitted with a 5-mm broad band probe. The ^15^N *T*_1_ was measured by recording a time series of ^15^N spectra using a 5° RF pulse every 3 min for 60 min and fitting the time evolution of the peak integrals to a single-term exponential decay model. The polarization was measured by comparing the integral of the signal in the first spectrum of the time series with the thermally polarized signal of a multiscan spectrum after addition of Omniscan gadolinium-based relaxation agent to decrease the long *T*_1_ of the compound. Analysis of spectroscopy data was done in Mnova (Mestrelab Research, Santiago de Compostela, Spain).

### Animals

All experiments were performed in accordance with the Animal Experiment Inspectorate in Denmark (permit number 2024-15-0201-01790). Three healthy female Wistar rats (244 ± 23 g) were anesthetized with isoflurane in O_2_ (1.5 to 2.5%). After cessation of reflexes, a tail vein catheter (24 G) was placed for administration of ^15^N,d_9_-betaine, and the animal was placed in prone position in an MR-compatible cradle. Temperature and respiration were continuously monitored and used to adjust isoflurane dose and animal heating, which was supplied via circulating heated water.

### MR hardware

MRI and MRS were performed on a clinical 3T MR system with multinuclear capabilities (SIGNA Premier, GE Healthcare). For proton imaging, a 21-channel receive-only head-and-neck-coil (GE Healthcare) was used.

For the ^15^N-acquisitions, a custom-made coil was used. The coil consisted of a transmit/receive (TX/RX) circular loop (80 mm in diameter) made out of solid copper wire (2.3 mm in thickness). It was tuned and matched to the ^15^N frequency at 3 T (12.95 MHz) using a simple balanced capacitive network with high-voltage rating ceramic capacitors (DLC70E, Dalicap, China). To switch between transmit and receive modes, a dedicated narrowband TR switch was developed, based on the design described by Thapa *et al.* (*68*).

The transmit path consists of a serial PIN diode (D1; MA4PK2000, MACOM, USA), which is biased during the transmit phase. The receive path consists of two Pi networks in series, each terminated by a PIN diode (MA4P7470F-1072T, MACOM, USA) in parallel to ground to improve the isolation. The lumped elements used to implement the Pi networks were *L* = 300 nanohenry (nH) and *C* = 503 pF. The TR switch was designed such that that all PIN diodes (in the TX and RX paths) are biased during transmission and reverse-biased during the reception. In addition, to protect the system preamplifier, a set of crossed diodes (UMX9989, Microchip) was added shunted at the output of the receive path.

The isolation and insertion losses of the TX/RX switch were measured on the bench using a vector network analyzer. The measured insertion losses in the receive path (with PIN diodes reversed-biased) was −0.86 dB. The measured isolation between transmit and receive was −39 dB in the receive state and −57 dB during the transmit state.

### *T*_1_ decay measurements at 3 T

To determine the *T*_1_ decay constant of hyperpolarized ^15^N,d_9_-betaine in buffer at 3 T and ~35°C, the dissolved sample (40 mM, 3 ml) was quickly transferred to the MR scanner where it was kept warm with water heating. The *T*_1_ decay constant was measured either by acquiring spectra for 16 min (flip angle = 1°, TR = 2 min, spectral bandwidth = 5000 Hz, 2048 spectral points) and fitting peak integrals to a single-term exponential decay model or by acquiring two spectra separated by 15 min (flip angle = 2°, spectral bandwidth = 5000 Hz, 2048 spectral points) and calculating *T*_1_ according to the signal decay within that interval.

To determine the *T*_1_ decay constant in blood at 3 T and ~35°C, the dissolved ^15^N,d_9_-betaine sample (40 mM) was transferred to the MR scanner where a 500-μ l sample was mixed with 2 ml of full blood (human) and kept warm with water heating. The *T*_1_ decay constant was measured either by acquiring spectra for 14 min (flip angle = 1°, TR = 2 min, spectral bandwidth = 5000 Hz, 2048 spectral points) and fitting peak integrals to a single-term exponential decay model or by acquiring two spectra separated by 17 min (flip angle = 5°, spectral bandwidth = 5000 Hz, 2048 spectral points) and calculating *T*_1_ according to the signal decay within that interval.

To estimate the apparent *T*_1_ decay constant in vivo, 1 ml of hyperpolarized ^15^N,d_9_-betaine (40 mM) was injected into the tail vein of three animals, and spectra were acquired from the abdominal region for 12 to 14 min (flip angle = 5°, TR = 2 min, spectral bandwidth = 5000 Hz, 2048 spectral points). Peak integrals were fitted to a single-term exponential decay model. A 10-Hz Gaussian filter was applied to all spectra, they were manually phase-corrected, and all analysis including fitting was done using MATLAB (version 2022b).

### Magnetic resonance imaging

The ^15^N coil was positioned posteriorly to cover the abdominal region of the animal (fig. S5A). Initial scout images were acquired using standard ^1^H three-plane fast gradient echo sequences. Subsequently, two-dimensional (2D) fast gradient echo imaging was performed in the coronal and sagittal planes for better planning of the ^15^N acquisition. Structural ^1^H scans were also acquired with a 2D fast gradient echo sequence in the axial and coronal planes from the same volumes as the ^15^N-acquisitions to provide anatomical references [TR/TE = 200/8 ms, flip angle = 20°, field of view (FOV) = 200 mm by 200 mm (axial) and FOV = 220 mm by 220 mm (coronal), matrix size = 280 × 280).

To assess the potential for multiple imaging experiments from a single hyperpolarized ^15^N,d_9_-betaine dose, we performed serial injections from the same dissolved sample, preserving portions unaffected by RF depletion. One dose of 2 ml (40 mM) was administered in two injections of equal volume (1 ml, injected over 10 s), followed by imaging on different timescales to capture both early and late distribution of the contrast agent. A single-shot 2D spiral readout (readout duration = 51 ms, matrix = 32 × 32, FOV = 80 mm by 80 mm) was used to acquire images from a 15-mm axial slice covering the kidneys [slice placement is indicated in fig. S5 (A and B)]. The first experiment was initiated immediately at the end of injection and lasted 30 s (flip angle = 10°, TR = 1 s), after which a 90° RF pulse was applied to deplete the remaining hyperpolarized signal. The second imaging experiment was initiated 5 s after the end of injection and lasted 12 min, using a variable flip angle scheme (flip angles = 3°, 5°, 7°, 10°, 15°, 24°, and 42°; TR = 2 min) to maintain near-constant signal throughout the acquisition.

From a second dose (1 ml, 80 mM), higher-resolution imaging in the coronal plane was performed, using a 2D spiral readout with three interleaves (readout duration = 67 ms, flip angle = 15°, matrix = 64 × 64, FOV = 80 mm by 80 mm, TR = 246 ms, 2 s additional delay between frames). Following injection (injected over 10 s), acquisition began immediately of a 15-mm slice covering the kidneys (slice placement is indicated in fig. S5B), with a temporal resolution of 2.74 s and five acquired frames (total scan time = 11.7 s). The GE MNS Research Pack (GE Healthcare, Munich, Germany) was used for the ^15^N MR sequence design.

### Image reconstruction and analysis

A 10-Hz Gaussian filter was applied along the readout dimension to enhance the SNR of the images. Images were zero-filled by a factor of two for visualization and reconstructed with standard gridding (*69*) and the inverse fast Fourier transform using MATLAB scripts from the GE MNS Research Pack (GE Healthcare, Munich, Germany). Correct center frequency was confirmed with slice-selective spectra acquired immediately following imaging from the same acquisition volume. ROIs including the vasculature and the left and right renal cortices were manually delineated from the center slice of the anatomical proton reference images, and the ^15^N,d_9_-betaine images were linearly interpolated to the proton matrix to generate signal time curves of summed signal within the ROIs. SNR was quantified using voxel signal intensities for images, or signal averages within a ROI, divided by the SD of signal intensities in a noise region outside the rat body (magnitude images, 2247 and 8988 voxels in noise region for low-resolution and high-resolution images, respectively). Image reconstruction and analysis were performed using MATLAB (version 2022b).
