# Supplementary material for: Dynamic, multiphase magnetic resonance imaging of in vivo physiological processes with long-lived hyperpolarized 15N,d9-betaine
Source: Sci Adv. 2025 Jul 2;11(27):eadx8417. doi: 10.1126/sciadv.adx8417 (PMC12219476; doi:10.1126/sciadv.adx8417)
Supplement: Supplementary file 1 — Figs. S1 to S5 [file sciadv.adx8417_sm.pdf]

Science Advances
AAAS

Supplementary Materials for

Dynamic, multiphase magnetic resonance imaging of in vivo physiological processes with long-lived hyperpolarized $^{15}N,d_9$-betaine

Ingeborg S. Skre *et al.*

Corresponding author: Mathilde H. Lerche, mhauler@dtu.dk

Sci. Adv. **11**, eadx8417 (2025)
DOI: 10.1126/sciadv.adx8417

This PDF file includes:

Figs. S1 to S5

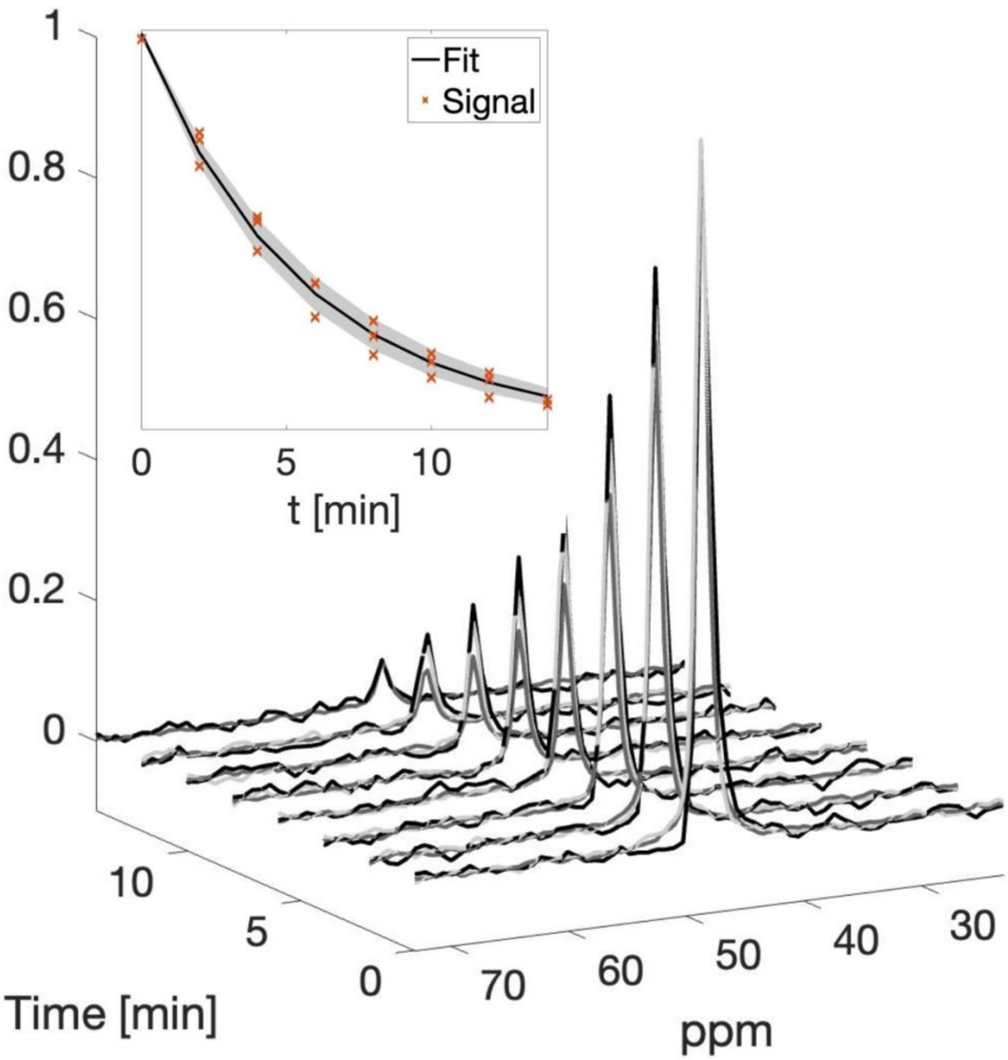

fig. S1

Normalized *in vivo* MRS spectra. Spectra were obtained of hyperpolarized $^{15}N,d_9$-Betaine at 3T, from the abdomen region of 3 rats. The spectra and integrals from all animals are displayed, together with the decay of integrals, where the solid line represents the average of the T_1 fits while the shaded area represents the standard deviation. The individual integral values are indicated by red crosses.

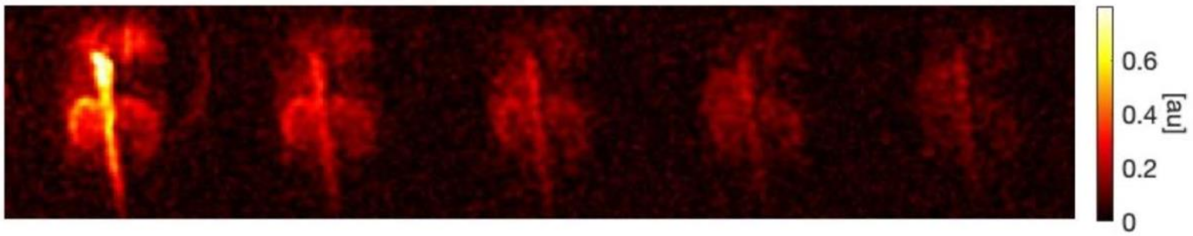

fig. S2

Higher resolution imaging of $^{15}N,d_9$-Betaine in the rodent kidneys in coronal view. Images are normalized to maximum intensity and colormaps of images are windowed to 80% of maximum signal.

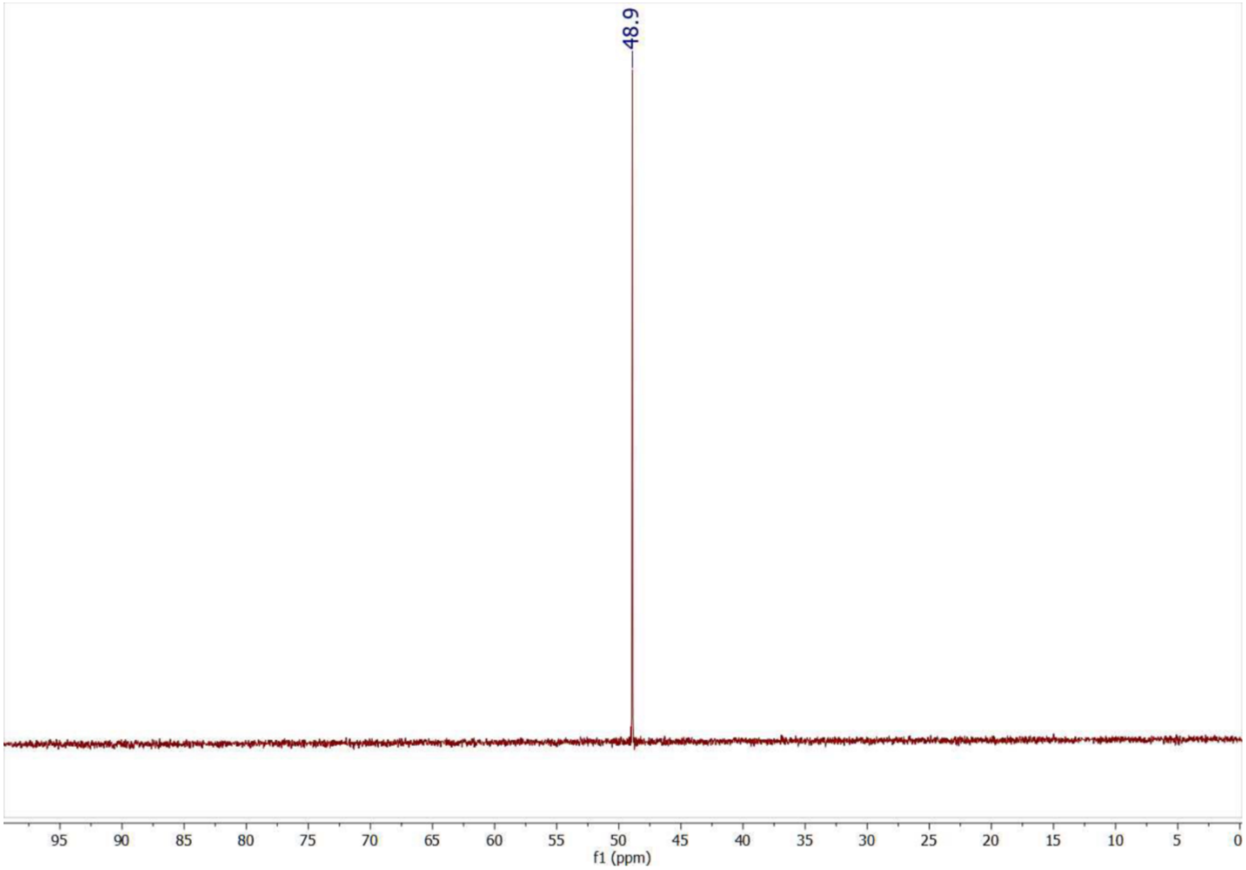

fig. S3

^{15}N NMR spectrum of $^{15}N,d_9$-Betaine. The spectrum was measured at 9.4 T on a Bruker Avance Neo spectrometer and referenced to ^{15}N-glycine at 33.5 ppm.

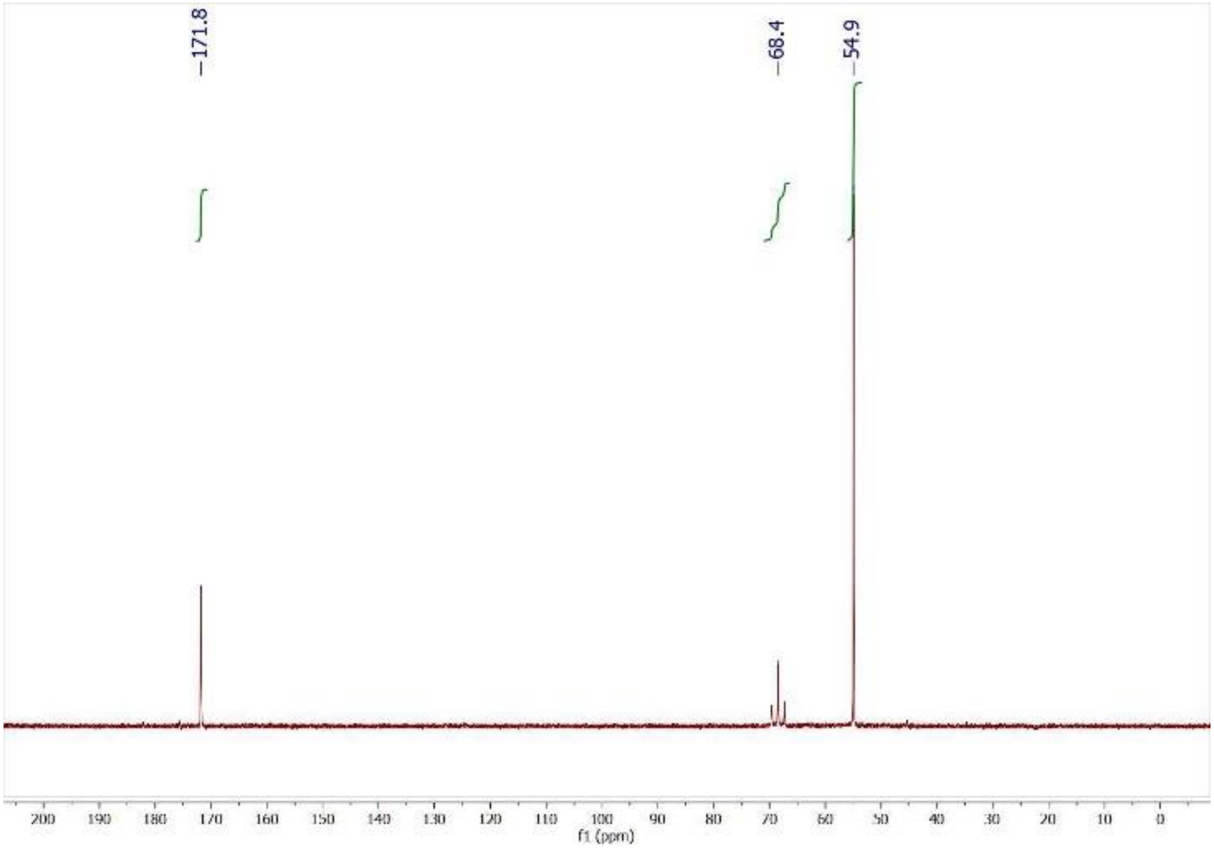

fig. S4

$^{13}C\{^{2}H\}$ NMR spectrum of ^{15}N,d$_9$-Betaine. The spectrum was measured at 9.4 T on a Bruker Avance Neo spectrometer.

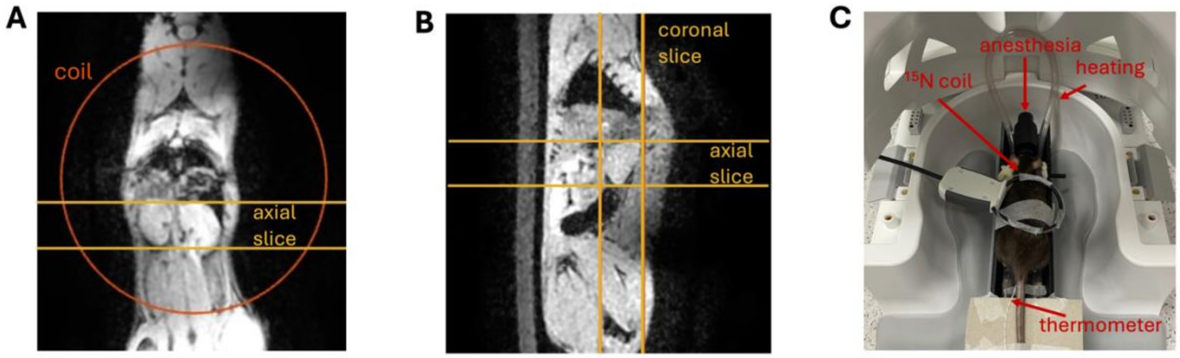

fig. S5

Set-up of *in vivo* ^{15}N-HypMRI experiments. (**A**) Placement of ^{15}N Tx/Rx loop coil and slice used for axial ^{15}N-imaging experiments (15-mm slice thickness) with reference to a ^{1}H image in coronal view. (**B**) Slice locations for axial and coronal ^{15}N-imaging experiments shown with reference to a ^{1}H image (a ^{1}H slice centered at the right kidney was chosen for display) in the sagittal view. Both axial and coronal slices were 15 mm thick. (**C**) Image of experimental set-up for in vivo imaging. The rat cradle is placed within a 21-channel ^{1}H head-and-neck coil.
